# When Does Overuse of Antibiotics Become a Tragedy of the Commons?

**DOI:** 10.1371/journal.pone.0046505

**Published:** 2012-12-07

**Authors:** Travis C. Porco, Daozhou Gao, James C. Scott, Eunha Shim, Wayne T. Enanoria, Alison P. Galvani, Thomas M. Lietman

**Affiliations:** 1 Francis I. Proctor Foundation for Research in Ophthalmology, University of California San Francisco, San Francisco, California, United States of America; 2 Department of Epidemiology and Biostatistics, University of California San Francisco, San Francisco, California, United States of America; 3 Center for Infectious Disease and Emergency Readiness, University of California, Berkeley, California, United States of America; 4 Department of Mathematics and Statistics, Colby College, Waterville, Maine, United States of America; 5 Department of Epidemiology, Graduate School of Public Health, University of Pittsburgh, Pittsburgh, Pennsylvania, United States of America; 6 School of Medicine, Yale University, New Haven, Connecticut, United States of America; 7 Department of Ophthalmology, University of California San Francisco, San Francisco, California, United States of America; 8 Institute for Global Health, University of California San Francisco, San Francisco, California, United States of America; University of Ottawa, Canada

## Abstract

**Background:**

Over-prescribing of antibiotics is considered to result in increased morbidity and mortality from drug-resistant organisms. A resulting common wisdom is that it would be better for society if physicians would restrain their prescription of antibiotics. In this view, self-interest and societal interest are at odds, making antibiotic use a classic “tragedy of the commons”.

**Methods and Findings:**

We developed two mathematical models of transmission of antibiotic resistance, featuring *de novo* development of resistance and transmission of resistant organisms. We analyzed the decision to prescribe antibiotics as a mathematical game, by analyzing individual incentives and community outcomes.

**Conclusions:**

A conflict of interest may indeed result, though not in all cases. Increased use of antibiotics by individuals benefits society under certain circumstances, despite the amplification of drug-resistant strains or organisms. In situations where increased use of antibiotics leads to less favorable outcomes for society, antibiotics may be harmful for the individual as well. For other scenarios, where a conflict between self-interest and society exists, restricting antibody use would benefit society. Thus, a case-by-case assessment of appropriate use of antibiotics may be warranted.

## Introduction

Over-prescribing of antibiotics has arguably led to an epidemic of drug resistant microbes [1] that increases morbidity and mortality among humans [2]. Thus, although antibiotic use may be beneficial to the individual, excessive use can be detrimental to the community. Limiting the use of antibiotics is predicted to address the problem [3], [4].

When the goals of the individual conflict with the goals of the community, a “tragedy of the commons” may result. Under such circumstances, individual incentives lead to the overuse and destruction of a shared resource, whereas restrictions to limit use would benefit all individuals [5], [6]. Classically, this is illustrated by the example of livestock grazing in a public commons. Each herder has an incentive to increase grazing as much as possible, yet if everyone does so, the land will be ruined. All would benefit from limiting access.

Does the emergence of drug resistance through antibiotic overuse constitute a tragedy of the commons as some authors have suggested [7], [8], [9], [10], [11]? Is treatment beneficial to individuals but harmful to society? Clearly, an increase in antibiotic selection pressure can increase the prevalence of resistance [12], [13].

This relationship has been demonstrated in theoretical models (e.g. [8], [14], [15], [16]), retrospective empirical studies (e.g. [17], [18]), and even prospective empirical studies (e.g. [19], [20], [21]). It is also generally accepted that resistance decreases the effectiveness of antibiotics (e.g. [22], [23]). However, the cost of this decreased effectiveness must be balanced against the benefit of reducing infections due to antibiotic-sensitive organisms. This trade-off has been investigated for the use of antivirals in controlling influenza in the population [24], [25], for instance. Of particular interest is the possibility that treatment of mild or less severe infection, while effective, may be unwise in part because of the development of drug resistance (e.g. [26], [27]).

In this paper, we analyze the conflict of interest between the individual and society using two compartmental models of treatment and drug resistance, and we assess when antibiotic use becomes a tragedy of the commons by analyzing the conflict of interest between the individual and society as a mathematical game. We also explore under what conditions antibiotic use becomes sub-optimal for society, despite the benefits to the individual. The models we chose are designed to address whether treatment of mild or early infection could constitute overuse of antibiotics. The results we derive are equally applicable in the more general setting of antimicrobial usage, and are not specific to antibiotics *per se*.

## Methods

### Overview

We analyze two simple compartmental models of disease transmission [28] and drug resistance. The first model includes transmissible drug-resistant strains or organisms and the development of resistance during treatment. The second model extends the first to include a mild early stage of colonization, infection or disease, and a more severe later stage of infection or disease. These models were designed to include features of HSV-2, tuberculosis, and pneumococcus (and other infections). Refinements of these models and application to specific infectious diseases is justified once an understanding of the dynamics of the simpler models has been obtained.

In both models, we proceed as follows. We first develop a Markov model representing the transitions of an arbitrary individual in the population. We then derive the corresponding population model by summing the state variables over all individuals. For the individual-level model, each individual may choose her or his own treatment rate for infection (in Model 1), or for mild infection (in Model 2). The payoff of each individual depends on not only the strategy chosen by that individual, but also on the choices of all other individuals insofar as those choices affect the overall forces of infection by drug-sensitive and drug-resistant organisms. After analyzing the outcome for an arbitrary individual subject to exogenous forces of infection, we then assume a large population, and sum the probabilities for each state to approximate the expected number of individuals in each state at the population level. At the population level, the forces of infection are not exogenous, but determined by the overall prevalence of drug-sensitive and drug-resistant organisms. For Model 1, we arrive at a standard deterministic compartmental epidemic model of SIS (susceptible-infective-susceptible) type [28] similar to previous models (e.g. [29], [15]), described by first order autonomous ordinary differential equations. A similar five-equation system describes the population for Model 2.

### Model 1

In Model 1, individuals are classified into three states: susceptible (uninfected), infected by drug-sensitive organisms, and infected by drug-resistant organisms. Two parameters govern the behavior of drug resistance: 1) the probability of generating a resistant infection during treatment of a drug-sensitive infection, and 2) the relative fitness of the drug-resistant organism. This simpler model is introduced in order to contrast its behavior with Model 2, below.

The force of infection (per capita hazard) for the drug-sensitive organism is denoted by 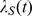, and the force of infection for the drug-resistant organism is denoted by 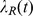. The mean duration of a sensitive or resistant infection is given by 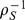 or 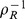, respectively. We assume that the rate of treatment for individual 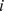 is 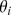, and that the probability of developing resistance during treatment is 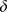. For each individual 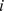, 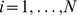, denote the probability of being susceptible at time 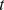 by 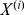, the probability of being infected by the drug-sensitive organism by 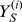, and the probability of being infected by the drug-resistant organism by 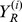. We describe the dynamics of the individual by the following three-state Markov chain:
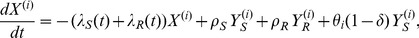
(1)

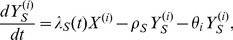
(2)and
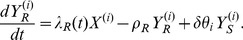
(3)Of course, 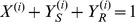 for all 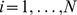. This yields 3N equations to describe the population.

We first apply this model to the experience of a single individual in a large population as in [30]. We assume constant forces of infection 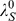 and 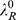; the risk of infection is determined by the population prevalence, and may be considered exogenous when modeling the experience of a single individual. We solved Equations (1), (2), and (3) for the equilibrium distribution of this Markov chain. From this equilibrium distribution, the equilibrium fraction of time spent in either disease state may be computed. Then, we may compute the optimal value of the individual treatment rate 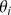 needed to minimize the fraction of time an individual spends in the disease state, given fixed 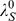 and 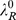. If, for particular values of the forces of infection and other parameters, an increase in 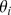 reduces the fraction of time the individual spends in the disease state (or, equivalently, increases the fraction of time spent in the uninfected state), the individual has an incentive to increase her or his treatment rate.

We next apply the model to the entire population as in [30]. Specifically, let 
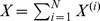
, 
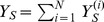
, and 
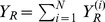
. Summing both sides of Equations (1), (2), and (3). This yields the following system:
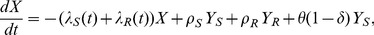
(4)

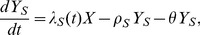
(5)and
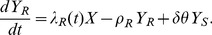
(6)where 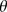 is an effective population-average treatment rate. Note that the total population size 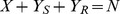 is constant.

We follow many other authors in assuming that the force of infection is proportional to the prevalence fraction (e.g. [31], p. 265; [28], [16], for a few of very many examples). Thus, at the population level the forces of infection are not exogenous constants, but are given by 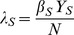 and 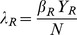, where, 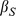 and 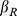 are transmission coefficients. Thus, changes in the population value of the treatment rate 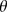 affect the overall equilibrium levels of disease, which in turn feed back and affect the forces of infection. Alternative functional forms, such as 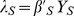 where 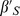 is constant (and similarly for 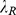), would yield a different functional dependence on population size 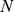.

For simplicity, we assume that an individual does not consider the side effects and cost when choosing treatment, and we assume that antibiotic supplies are not limited. We assume that the transmission coefficient of a drug-resistant organism is no more than that of a drug-sensitive organism, because otherwise the drug-resistant organism would presumably have predominated even before antibiotic use, so that 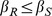. In fact, resistant strains may be less transmissible due to a fitness cost of resistance [32], [33], which we have expressed as a lower transmissibility (transmission coefficient); it is straightforward to extend analysis to alternative models for relative fitness, such as a reduced duration of infectivity (e.g. [34]), though such analysis is not presented in this paper.

Equations (4), (5), and (6) constitute a standard two strain compartmental epidemic model of SIS type (e.g., [35], [28]); the dynamics of competitive exclusion in such models is well understood in a more general demographic setting (e.g. [36], [37]), and dynamic control of a two strain SIS model without acquired resistance has been explored [38]. The equilibrium fraction of the population in the diseased states is computed below, as well as the value of the population treatment rate 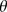 which minimizes this fraction. The latter value is the utilitarian optimum value of the treatment rate at the population level. Analysis was performed with the assistance of the computer mathematics package SAGE [39].

Model 1 may be analyzed as a dynamic stochastic 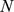-player game, because each individual may choose a strategy 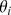, i.e. a particular value of the treatment rate. If an infected individual chooses a treatment rate of 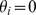, she or he is never treated, and if infected, suffers the course of the disease and may spread the infection to others. The payoff to the individual is the fraction of time spent healthy (in state 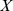). However, this payoff is determined not only by her or his choice of treatment strategy. It is also determined by the choices made by all other individuals in the population, which together determine the forces of infection. This game is analyzed by assuming a given level of population treatment and the forces of infection implied by the choice. We determined whether or not a given individual in the population has an incentive to deviate from the population choice, i.e. can the individual reduce his or her level of disease by choosing a value of the treatment rate that differs from that of the population? A value of the population 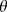 such that any individual who deviates from it will achieve a lower payoff constitutes a Nash equilibrium, regardless of whether or not this value coincides with the utilitarian optimum.

### Model 2

We extend Model 1 to include a mild state of colonization, infection or disease, which may or may not progress further to a severe state. Model 2 is a compartmental model designed to reflect three relevant features of infectious diseases. As in Model 1, the model includes the **development of resistance during treatment** (either by mutation or by the acquisition of resistance factors), as seen, for example, in tuberculosis [40], HSV-2 [15], or HIV (e.g. [41], [42], [14]). However, unlike Model 1, the disease exhibits a **spectrum of clinical severity**, with a milder form which may be followed by a more severe form. The treatment is the same for the mild and the severe forms. For example, antibiotic treatment may eliminate pneumococcal disease and temporarily eliminate pneumococcal colonization (e.g. [43]); though one would not ordinarily treat colonization, the presence of antibiotics applied for other reasons (e.g. [44], [45]) may nevertheless affect colonizing organisms. HIV (e.g. [46]) provides another example of progression along a spectrum of severity (in this case, without recovery). Finally, as in Model 1, the model requires the possibility that **drug resistant organisms be transmissible from person to person**, consistent with the biology of HIV (e.g. [47], [48], [49]), tuberculosis (e.g. [50], [51], [52]), and HSV-2 [15], among many others. In the discussion of this model, we will always refer to “mild infection”, with the understanding that this state refers simply to an earlier state of colonization or infection which may or may not be treated. We do not explicitly model mortality or health state utility in Model 2, but assume that the goal of individuals is to minimize the amount of time spent in the second, severe, states.

When the infection is caused by a drug-sensitive organism, treatment may be applied in the mild state. This treatment may cure the infection and thereby prevent progression to a severe state. An individual with mild infection may clear the infection without ever entering the severe state, and so we may ask whether or not treatment of this state is desirable—whether such treatment could constitute overuse of antibiotics. In any event, treatment may fail due to the development of drug resistance, allowing the mild infection to progress to severe infection. Treatment may also be applied in the severe state. The treatment may cure the disease at this stage provided the organism is drug-sensitive, or fail with the development of drug resistance. We assume treatment is unsuccessful for drug-resistant organisms whether in the mild or severe state.

We analyze the question of what treatment rate should be chosen for the mild state. Severe disease, we assume, will always be treated at some given rate regardless of what treatment rate is chosen for mild infection. Note that for Model 1, the payoff is the fraction of time spent uninfected, and the control variable is the treatment rate. For Model 2, the payoff is defined as the fraction of time spent without severe disease, and the control variable is the treatment rate for mild infection. The fraction of time that an individual spends in a *severe* state (whether sensitive or resistant) was computed for each level of treatment of the mild stage the individual chooses. Maximizing the individual's time spent outside the severe states is of course the same as minimizing the time spent in a severe state (whether resistant or sensitive).

As for Model 1, the analysis for Model 2 consists of two stages: (1) analysis of the best strategy for an individual faced by constant exogenous forces of infection (unaffected by the treatment rate for mild infection that the individual chooses), and (2) analysis of how the population fraction of time spent in the severe state is minimized by the choice of treatment rate for mild infection. We determine whether or not an individual has an incentive to treat mild infection more or less than the other members of the population with mild infection, in the same way as we analyzed Model 1.

For Model 2, we assume that each individual 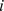 in the population (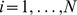) may be either susceptible, or may have mild infection with drug-sensitive organisms, mild infection with drug-resistant organisms, severe infection with drug-sensitive organisms, or severe infection with drug-resistant organisms. The probabilities that individual 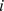 is in each of these states are given by 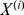, 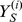, 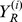, 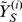, and 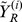, respectively. Individual 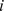 is assumed to choose treatment rate 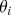 for the mild state. Individuals with mild infection progress to severe infection at a constant rate; treatment of the mild state is modeled as a competing exponential risk. We assume constant hazards for recovery from infection in all cases. Specifically, 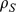 and 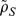 denote the recovery rates from mild and severe infection with the drug-sensitive organism, respectively, and 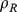 and 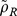 denote the recovery from mild and severe infection with the drug-resistant organism, respectively. We assume constant rates 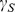 and 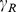 of progression from mild to severe infection with sensitive and resistant infections, respectively, and we assume 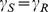. The antibiotic treatment rate for severe infections is denoted by 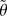, and this is assumed to be the same for everyone regardless of what choice is made for mild infection. We assume acquired resistance probabilities of 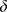 for mild infection and 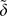 for severe infection. Finally, denoting the force of infection with the drug-sensitive organism given by 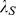 and the force of infection with the drug-resistant organism given by 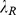, the dynamics of individual 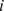 is given by the following five state irreducible ergodic Markov chain:
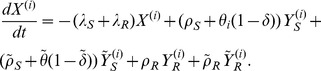
(7)

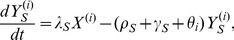
(8)

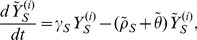
(9)

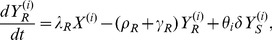
(10)and
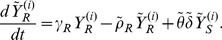
(11)


Each individual may choose a different strategy 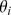, and the payoff to individual 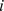 is determined by her or his choice of strategy, but also by the choices of all the other individuals in the population. Similar to Model 1, these equations represent an 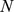-player game. As emphasized earlier, in Model 1, the individual payoff was the fraction of time spent healthy, and the strategy was the treatment rate of infection, while in Model 2, the individual payoff is the fraction of time spent without severe disease, and the strategy is the treatment rate of *mild* infection.

**Figure 1:**
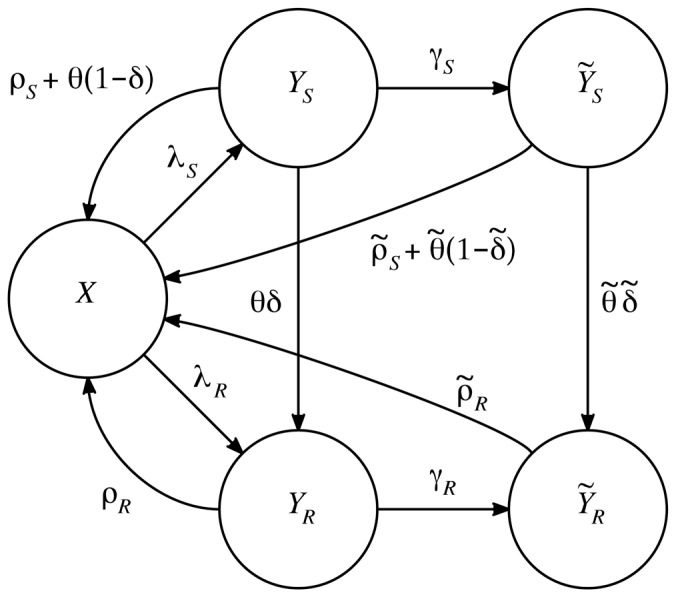
Compartmental flow diagram for Model 2. Each circle represents a state variable; each arrow a transition. The state variables are: 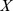
—the number of uninfected individuals, 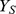
—the number of individuals with mild infection by the drug-sensitive organism, 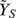
—the number of individuals with severe infection by the drug-sensitive organism, 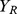
—the number of individuals with mild infection by the drug-resistant organism, and 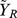
—the number of individuals with severe infection by the drug-resistant organism. Treatment rates for the mild and severe state are given by 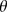 and 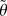, respectively. The arrows are labeled with per-individual flow rates; the total flow rate from each state along each arrow is given by the label of the arrow times the number of individuals in the state. The explicit differential equations and parameter definitions are given in the main text.

We define 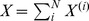, 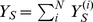, 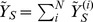, 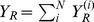, and 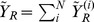. We sum over both sides of Equations (7), (8), (9), (10), and (11), yielding the following deterministic ordinary differential equations (see Figure 1). For susceptibles,
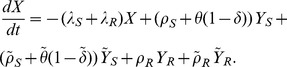
(12)For mild sensitive infections,
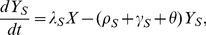
(13)and for severe sensitive infections,
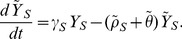
(14)Similarly, for mild resistant infections,
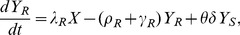
(15)and for severe resistant infections,
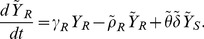
(16)Here, 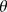 is an effective population-average treatment rate. We define the payoffs in terms of the equilibrium solutions of this system first in the individual setting in which we assume exogenous forces of infection, and then in the community setting. Analysis was performed with the assistance of the computer mathematics package SAGE [39].

## Results

### Model 1

#### Individual dynamics

To apply Model 1 to a given individual in the population, we compute the equilibrium fractions of time spent in each state, denoted by a superscripted *, using Equations (1), (2), and (3), based on the treatment rate 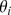 one specific individual. For a single individual, the forces of infection are determined by the choices of the population and are unaffected by the choice of any single person. When modeling a single individual, the forces of infection 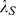 and 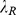 are functions of the community treatment rate (and will be discussed separately), but are exogenous, *i.e.* unaffected by the decision of that single individual; we write 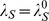 and 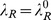 to emphasize this. Setting the left hand sides of Equations (1), (2), and (3) to zero yields
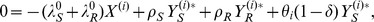


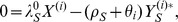
and
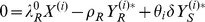
(with one equation being redundant, since 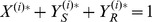 for all 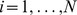). We find that the fraction of time spent in the susceptible state is
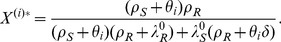
(17)It can be shown that 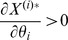 if and only if 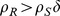. When the two diseases are assumed to have the same duration of infection, this inequality reduces to 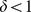; provided that treatment is not certain to fail (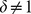, i.e. 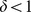), an individual always benefits from increasing treatment.

#### Community dynamics

For the community, the forces of infection for drug-sensitive and drug-resistant organism are not exogenous. Using Equations (4), (5), and (6), and setting 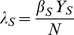 and 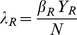, gives a conventional epidemic model of SIS type. We denote the basic reproduction number of the sensitive organism as 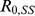, which is the number of secondary cases an initial drug-sensitive infective can cause in a completely susceptible population, in the absence of treatment; 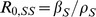. When treatment is undertaken, the reproduction number of the drug-sensitive organism is denoted by 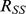; when the treatment rate 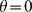, this treated reproduction number specializes to 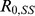. Equations (4), (5), and (6) imply that 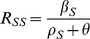, and that the equilibrium fraction of susceptibles is 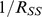 when 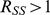, or 1 when 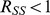. When 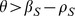, 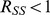, and the drug-sensitive organism is eradicated. The reproduction number of the drug-resistant organism can be shown to be 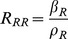. In general, it can be shown that (i) if 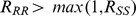, the drug-resistant organism competitively excludes the drug-sensitive organism at equilibrium, (ii) if 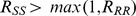, the endemic equilibrium features coexistence of both drug-sensitive and drug-resistant organisms provided 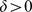 and 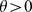, and (iii) if 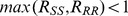, disease does not persist at equilibrium.

We denote the equilibrium values of the total number of uninfected individuals by 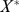, and for the number of individuals with mild drug-sensitive infection, severe drug-sensitive infection, mild and drug-resistant infection by 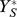, and 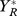., respectively. In this model, when 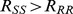 and 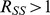, we find that the population payoff (fraction not infected) is given by
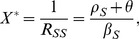
(18)a result that does not depend on either 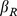 or 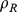. In Model 1, the larger the value of 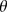 at the community level, the lower the fraction infected. Provided
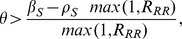


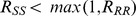
. Thus, it is always possible to eradicate the drug-sensitive organism, provided a sufficiently high treatment rate in the mild state can be achieved. When 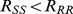 and 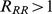, the drug-resistant organism competitively excludes the drug-sensitive organism, 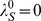, 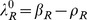, and 
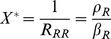
. Further increases in the treatment rate play no role, *i.e.* the equilibrium values are not changed by increasing the treatment rate above the critical value needed to eradicate the drug-sensitive organism, since the equilibrium prevalence of infection by the drug-sensitive organism is zero. Equation (18) implies that 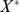 is continuous as 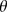 increases through the critical value 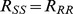.

Finally, the value of 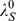 at the coexistence equilibrium for Equations (4), (5), and (6) is given by
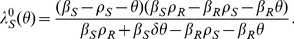
(19)Similarly,
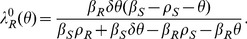
(20)The optimum treatment level for the community is the smallest treatment rate needed to eradicate the sensitive organism; 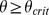, where 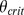 is the value of 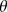 such that 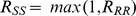. If in addition 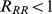, then the population treatment rate is sufficient to eradicate the infection; 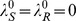. This conclusion depends on the assumption that 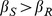, implying 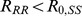; if 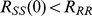 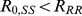 and 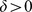, the drug-resistant organism will outcompete the drug-sensitive organism once it is introduced into the population.

#### Mathematical game

For a given set of parameters, the best choice for any given individual is to treat at either a rate of zero, or at the highest possible rate (depending on 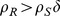). Because each individual in the population has the same choice, we can represent the game by the 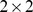 normal form shown in Table 1. Each row represents a decision by a particular individual, and each column represents a unanimous decision by the rest of the community. The cells show the welfare or payoff of an individual, given by 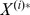 (Equation 17). To compute the payoff in each cell, we substitute the individual value of 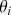 into Equation 17, and we solve for 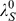 and 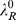 given the community level of 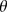.

**Table 1:** Antibiotic use modeled as a mathematical game.

Individual chooses treatment	Payoff 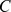	Payoff 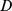
	Infection risk elevated because everyone else chooses no treatment; individual suffers the full course of resistant infections only	Infection risk reduced because everyone else chooses treatment; individual suffers full course of resistant infections only

Each row corresponds to the strategy of a particular individual, and each column corresponds to a unanimous strategy chosen by the rest of the population. The welfare or utility of the individual player is represented in each cell (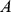, 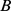, 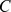, or 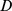), and can be calculated directly from Equation 17 by substituting the individual's choice of treatment rate 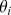 and the forces of infection 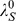 and 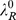 resulting from the community choice of 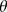. See Text S1 for details.

What is implied by this table? Here, 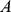 is the payoff when both the individual and everyone else chooses no treatment (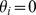, and 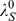 and 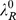 are given by their equilibrium values given the community treatment rate); similarly, 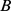, 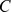, and 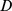 are payoffs as given in the table. Whenever 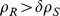, we know that treatment benefits the individual, so that 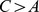 and 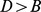 (whatever forces of infection 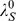 and 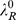 result from community treatment). As long as there is some risk of being infected by a sensitive strain, an individual benefits from choosing to be treated, regardless of whether everyone else chooses to be treated; it is always to the advantage of the individual to be treated, because the infection may be sensitive and there is no disadvantage to treatment (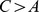 and 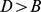). In the Text S1, we show that 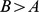. Thus, 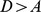, and in this simple case, society does not suffer from exploitation of the antibiotic. If everyone is treated, the prevalence of drug-resistant infection is higher, but this is offset by the reduced prevalence of drug sensitive infections. In Model 1 (though not in Model 2), there can be no tragedy of the commons resulting from overuse of antibiotics (Table 1). When 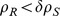, or equivalently 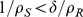, it becomes possible for treatment to benefit society even though it is harmful to the individual (see Text S1).

### Model 2

Our analysis of Model 2 is similar to that of Model 1, except that for Model 2 we analyze the treatment rate of mild infections. We derive two outcome variables: 1) the proportion of time an arbitrary individual would spend in severe infection states, and 2) the overall prevalence of infection in the population.

#### Individual dynamics

We determine the criterion under which increasing treatment of the mild state causes an individual to spend more time in the severe state. We assume that the decision of any particular individual to treat mild infection has no effect on the overall population forces of infection for resistant and sensitive infections, so that the forces of infection are exogenous. Equations (7)–(11), with constant forces of infection, describe a five-state Markov chain in continuous time, representing the state transitions of individual 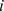 in the population, given these exogenous and constant forces of infection.

Let 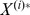, 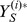, 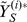, 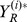, and 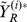 denote the equilibrium probabilities for the five states for individual 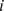. These solutions may be expressed in terms of 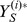, so that
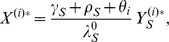


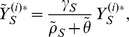


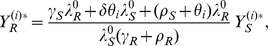
and
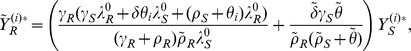
where 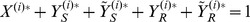.

The fraction of time an individual spends in the severely diseased compartment is a function of the treatment rate for mild infection 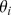 chosen by the particular individual 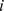, and by the average community treatment rate 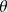 for mild infection:
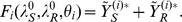
Note that each individual has her or his own payoff function; the dependence between individuals is introduced solely through the epidemic process in such a way that at equilibrium, the rest of the population's choices affect individual 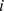 solely in terms of the average treatment rate in the community, 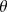. Substituting in the above expressions and canceling factors of 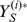 yields the following expression for this fraction of time:
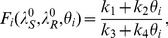
(21)where the values of 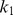, 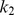, 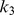, and 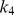 depend on 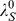 and 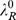, and cannot be negative:
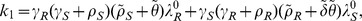


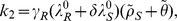


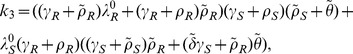
and
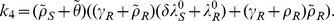
The denominator of Equation (21) can only vanish for negative 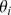, and so the function 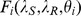 must be monotone increasing for all nonnegative 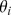 whenever 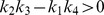. Similarly, the denominator is monotone decreasing when 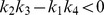. When 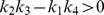 and 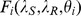 is therefore monotone increasing for positive 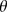, the higher the treatment rate for individuals in the mild state, the more time they experience in the severe state. In this case, the optimum level of treatment for an individual is zero. Similarly, when 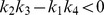 and 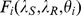 is thus monotone decreasing, the larger the rate of treatment of individuals with mild infection, the less time spent in the severe state. In this case, the individual's optimum treatment level is the maximum possible treatment rate. Thus, given specified exogenous values for the forces of infection, we can determine if increasing the treatment rate for mild infection benefits the individual by computing the sign of 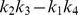. Substituting into 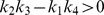 and rearranging, we find that increasing treatment causes the individual to spend more time in the severe state when
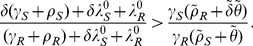
(22)Thus, the optimum strategy for any individual is to either not be treated at all in the mild state, or to be treated at the maximum possible rate, depending on condition (22).

When the resistant and sensitive infections have the same progression and recovery rates (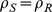, 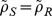, and 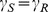), condition (22) reduces to
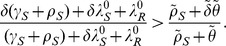
If we assume that the severe state cannot be treated (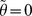), the entire expression reduces to the impossibility 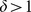. Thus, when the natural histories are identical for sensitive and resistant infections, the possibility that more treatment can harm the individual arises entirely from lost treatment opportunities in the severe state. Because increasing the treatment rate can harm the individual, it is possible that increasing the treatment rate could harm the community, raising the question of whether or not the individual incentives always match the community incentives.

#### Community dynamics. Competitive exclusion

At the population level, the forces of infection are determined by the prevalences of infection due to drug-sensitive and drug-resistant organisms. Changes in the treatment rate of mild infection at the population level affect the force of infection and prevalence of infection. For this calculation, we assume that the force of infection is a linear function of the prevalence fraction of both mild and severe infection. Denoting the transmission coefficient for individuals with mild infection due to the drug-sensitive organism as 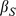 and the transmission coefficient for individuals with severe infection due to the drug-sensitive organism as 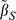, we let 
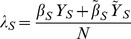
 be the force of infection for the drug-sensitive organism. Similarly, we denote the transmission coefficient for individuals with mild infection due to the drug-resistant organism as 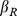 and the transmission coefficient for individuals with with severe infection due to the drug-resistant organism as 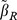. We let 
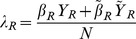
 be the force of infection with the drug-resistant organism.

In this case, the system exhibits three equilibria: (1) the no-disease equilibrium, (2) a resistance-only equilibrium, and (3) a coexistence equilibrium. The behavior of this system is qualitatively the same as seen in other models (e.g. [53], [54]), and we omit details. The next generation matrix [55] is
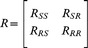


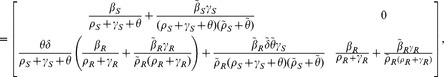
where 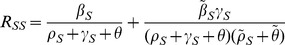, 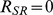, 
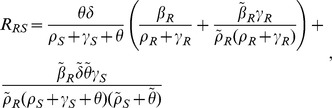
 and 
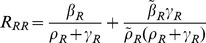
.

When 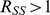 or 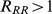, the no-disease equilibrium is unstable. When 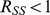 and 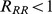, the no-disease equilibrium is stable and the endemic equilibrium does not exist. Also, 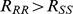 and 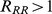 together imply that the drug-resistant organism competitively excludes the sensitive organism (no stable coexistence equilibrium exists). Finally, 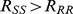 and 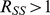 implies that the coexistence equilibrium is stable and the resistance-only equilibrium is unstable. In particular, the drug-sensitive organism does not exclude the drug-resistant organism, because treatment continually produces new drug resistance (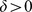 or 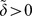).

We assume that all severe infections progressed from mild infections, and so in the limit 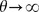, 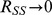. Thus, when the treatment rate for the mild state satisfies
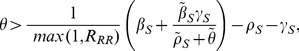
(23)the drug-sensitive organism will always be eliminated. If 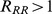, then the drug-sensitive organism is competitively excluded for values of 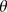 above the critical value for which 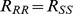.

The equations yield explicit values for the equilibrium fractions of the population in the states corresponding to severe infection (by drug-sensitive and drug-resistant organisms). These fractions are shown in Text S1, and from them, the equilibrium prevalence of severe infection can be computed. In this model, harm to society is measured as the prevalence of severe infection, and harm to an individual is measured by the fraction of time an individual spends in the severe state, given exogenous forces of infection for the drug-sensitive and drug-resistant organisms.

Assuming that 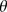 is large enough that 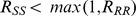 as shown in Equation (23), we have two cases: 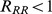 and 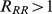. In the case 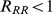, the drug-resistant organism cannot cause a self-sustaining endemic. The optimum strategy for the population is to choose a treatment rate for mild infection large enough to eradicate the sensitive organism, though such a treatment level may not be feasible. Considerations of cost, not included in this model, would suggest that the smallest such treatment rate be chosen. In the case 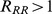, it is possible to increase the treatment rate of mild infections such that 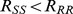, at which point the drug-resistant organism excludes the drug-sensitive organism entirely.

#### Optimal strategies

**Figure 2:**
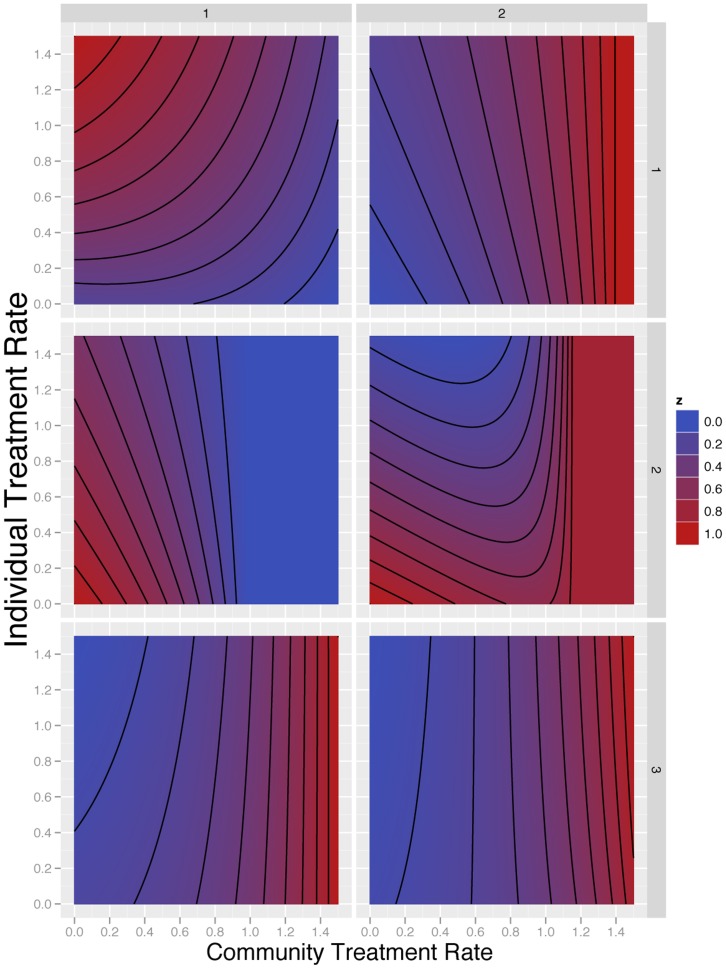
Relative fraction of time spent in the severe disease under six different scenarios. The 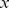-axes are the community level of treatment, i.e. the strategy assumed chosen by all other members of the community. The 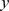-axes are the level of treatment chosen by an individual within the community. The contour plot shows the fraction of time spent by this person, in the severe state; each panel has been scaled so that the minimum value is zero (blue) and the maximum value is 1 (red). The numerical parameter choices are given in Table 2, and the minimum and maximum values for each panel.

Assuming each member of the community chooses treatment rate 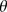 for the mild state, we assesse the fraction of time spent in the severe stage for any individual choosing a different strategy from that of the community, i.e. treatment rate 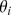 for the mild state. According to Equation (21) (and Equation (7) in Text S1), different parameter choices in Model 2 lead to very different game theoretic outcomes, as shown by specific numerical examples. We present six scenarios to illustrate the behavior of 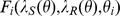 as given by Equation (21) with 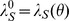 and 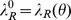 given by Equations (5) and (6) in Text S1. These are as shown in Figure 2. Each scenario corresponds to a different set of parameter values, given in Table 2; these scenarios were chosen to illustrate the range of behavior implied by Equation (21) and have no spcecial significance *per se*. The strategy chosen by the specific individual of interest, 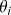, is given on the vertical axis, while the community level of treatment 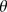 is shown on the horizontal axis. For each set of parameter values, we computed the fraction of time an individual spent in the severe state.

**Table 2:** Numerical scenarios for Figure 2.

Panel	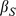	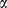	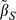	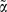	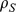	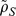	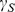	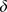	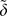	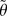	Min	Max
Upper Left	4.31	0.111	3.63	0.267	0.85	0.91	1.38	0.53	0.01	3.86	11%	23%
Upper Right	4.91	0.493	3.9	0.469	1.64	0.2	0.03	0.19	0.08	3.24	1.4%	5.0%
Center Left	3.25	0.563	3.8	0.576	1.21	1.64	0.16	0.25	0.24	0.75	2.9%	4.2%
Center Right	4.19	0.544	3.99	0.226	0.67	1.42	0.86	0.07	0.33	3.02	15%	18%
Lower Left	5.51	0.318	6.6	0.127	1.27	0.38	2.09	0.04	0.01	2.84	27%	34%
Lower Right	6.96	0.353	6.88	0.766	1.01	2.98	0.36	0.15	0.14	3.95	4.7%	5.4%

In all scenarios, 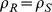, 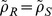, and 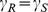. The first column refers to the panel in Figure 2. Subsequent columns give the particular parameters chosen for the panel. The final two columns provide the minimum equilibrium prevalence and the maximum equilibrium prevalence of the severe state (from Equation (7) in Text S1), respectively, for the parameters in the panel.

The upper left panel of Figure 2 illustrates a scenario in which increasing treatment of the mild state generates more resistance for any particular individual, whatever the community has chosen to do. Beginning at community treatment rate 0, an individual who increases his or her treatment rate in the mild state will spend more time severely ill, due to acquiring drug resistance and being unable to treat resistant severe infection. The highest payoff that can be achieved by any particular individual occurs when the individual chooses no treatment in the mild state, but the rest of the population chooses a high treatment rate for the mild state.. In this scenario, treatment of others reduces the overall prevalence of disease, and a given individual may gain this benefit without taking the risk of treating mild infection themselves.

A different scenario is illustrated in the upper right panel. In this scenario, an individual who chooses to increase her or his treatment rate in the mild state always attains a lower payoff as a result. Moreover, the rate of severe disease increases in the community as the community rate of treating the mild state increases. This scenario shows no divergence of individual and community incentives. Individuals who choose to treat the mild state are overusing antibiotics, and if the community increases the rate of antibiotic use in the mild state, the entire community achieves a lower payoff.

The center left panel of Figure 2 is a scenario in which both the individual and the community benefit from increased treatment of the mild state. For the parameters chosen here, increasing treatment of the mild state yields increasing drug resistance. However, the drug resistance is outweighed by the reduction in overall disease that results from treatment.

For low treatment rates, the scenario on the center right is qualitatively similar to the scenario on the center left. However, in the center right scenario, at higher community treatment rates for the mild disease, infection is not eliminated. The drug-resistant organism eventually competitively excludes the drug-sensitive organism, and the community achieves a less favorable outcome.

In the bottom left panel, individuals always benefit from increasing their treatment level in the mild state, regardless of the community treatment rate; whatever the community chooses, it is always better for an individual to increase her or his treatment rate. However, for a given treatment rate of mild infection an individual chooses, an individual spends more time in the severe state if the community treatment rate increases. In this scenario, individuals who increase their treatment rate for the mild state spend less time in the severe state. Unfortunately, as community rates increase, so does the overall community prevalence of severe disease. Individual incentives are not aligned with community welfare. Unlike the overuse scenario from the upper right, the lower left panel describes a tragedy of the commons.

The scenario in the lower right is similar to the previous scenario for low treatment levels. At low community levels of treatment, an individual benefits by choosing increased treatment of the mild state. However, if the community treatment levels are higher, the individual benefits by treating the mild state at a lower rate than the community average treatment rate for mild infection. The community overall achieves a less favorable outcome if all choose higher treatment rates for mild infection. This effect occurs because at high treatment levels of mild infection in the community, the force of infection for the drug-resistant organism is large enough that individuals who clear the drug-sensitive organism are soon reinfected by the drug-resistant organism. Treatment rates corresponding to the classical game of *Chicken* ([56], p. 18) may be derived.

**Figure 3:**
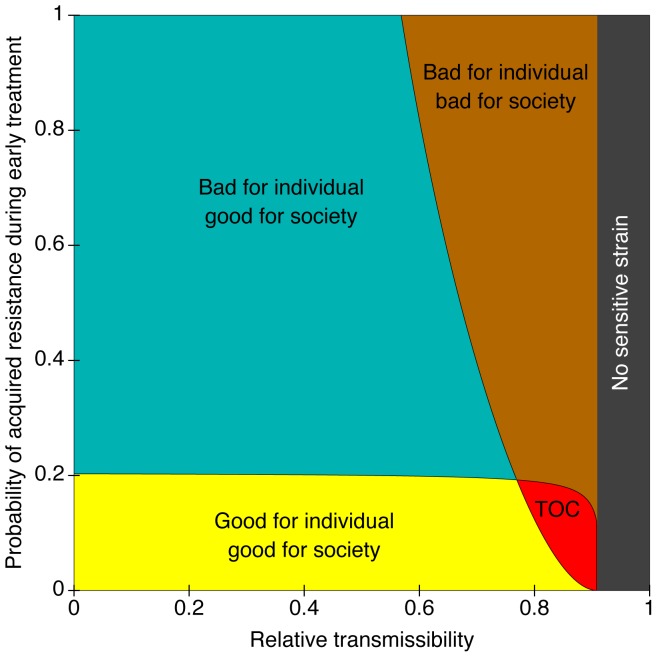
Assessment of the effect of over-treatment of mild infection on the treatment of mild infections. It was assumed that severely infected individuals do not transmit, and that drug resistance does not develop during treatment of severe infections. The mean time to treatment is set at 5 (arbitrary units) for mild infection, and 1/3 units for severe infection. All other expected waiting times (recovery, progression) are equal to 1. The reproduction number for the drug-sensitive organism is 1.5. See Text for full details of Model 2. Under these assumptions, the drug-resistant organism competitively excludes the drug-sensitive organism whenever the relative transmissibility exceeds 10/11 (91%) (grey area, labeled “No sensitive strain”). The parameters are 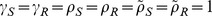, 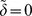, 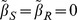, 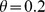, 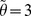, 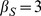, and 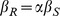. The horizontal axis corresponds to 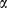 and the vertical axis to 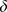.

Another approach to the tradeoffs between the individual and society is obtained if we assume a given community treatment rate, and then examine whether an individual should deviate infinitesimally from that rate. Similarly, we can ask whether or not the community should increase or decrease the overall rate of mild treatment by a small amount. In essence, we are considering small 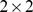 games in which the community strategy is to stay the same, or to change the treatment rate for mild infection, and the given individual strategy is to stay the same or to change the treatment rate of mild infection for themselves. An example is shown in Figure 3. Five possible outcomes are obtained, depending on how frequently resistance occurs *de novo*, and depending on the relative fitness of a drug-resistant organism. 1) As in Model 1, increasing antibiotic use may benefit the individual and society (yellow region). 2) A second possibility is that increasing antibiotic use in the mild state is harmful for the individual, but good for society. This may occur when drug-resistance is likely to be acquired *de novo*, but infrequently spreads from person to person (blue region). In this case, treatment of the mild disease is harmful to the individual because the development of resistance makes it difficult to treat the severe disease which may develop later. Reduced transmissibility of the drug-resistant organisms means less infection is spread to others. 3) When resistance is easily acquired and frequently transmitted, increased antibiotic use may be harmful to both the individual and society. 4) When resistance occurs infrequently and is likely to be transmitted, a tragedy of the commons is possible (red region). 5) Finally, if the pathogen pays little or no fitness cost for resistance, the drug-sensitive organism may become extinct due to competitive exclusion, and any further treatment is irrelevant to both the individual and society (gray vertical region on the right). In this model, increased antibiotic use may benefit society in which case antibiotic restrictions would not be warranted. However, treatment of mild disease may cause more harm than good if competitive exclusion of the drug-sensitive organism results.

## Discussion

In this study, we develope a simple transmission model of the development and spread of drug resistant organisms, and assess the difference between individual and community incentives. The model assumes infection or disease is classified into an early milder stage and a later, more severe stage; treatment may occur in either stage. In our model, drug resistance may develop during treatment and may be transmitted. We analyzed the conflict of interest between the individual and the community using the fraction of time spent in a severe state to define an objective function to be minimized. Assuming that each individual may vary his or her individual treatment rate allows the problem to be treated as an 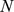-player game. We analyzed the static dynamics of this game to show that antibiotic use may indeed lead to a tragedy of the commons [7], [8], [9], [10] in which individual incentives lead to antibiotic use rates that are too high to yield the best community outcome. Other parameter values lead to other results; a tragedy of the commons resulting from overuse does not always result (and never results in the simpler model we examined).

In a tragedy of the commons, the goals of society are in fundamental conflict with the goals of the individual. Such conflicts are well documented, such as for vaccination against now-rare diseases [57], [58], [59]. In these previous studies, vaccination eventually reduces the prevalence of infection to such low levels that the harm expected to result from adverse outcomes of vaccination exceeds the expected benefits of vaccination. When this happens, the optimal decision for each individual is to forego vaccination. Subsequent failure of a substantial fraction of the population to become vaccinated may then allow the resurgence of disease. Conflicting interests exist in influenza control as well. Widespread use of antiviral drugs for treatment and prophylaxis of influenza may lead to a high prevalence of resistant organisms even when the probability of developing resistance during treatment is small. Thus, although individuals are compelled to seek out treatment, society as a whole may suffer from increased circulation of resistant organisms as a consequence of individual treatment [24]. However, the spread of resistant organisms may be contained if control measures are taken fast enough to contain the initial outbreak of sensitive virus [25]. Even if strong control measures are implemented and successful, the population may be at risk for experiencing a largely uncontrolled subsequent outbreak [60]. A different sort of conflict of interest arises when treatment of HIV/AIDS increases life expectancy, thereby prolonging the infectious period [61], though increased opportunities for transmission are arguably outweighed by the reduced infectivity due to lowered viral loads (e.g. [62]).

For the case of drug resistance, it is not difficult to produce scenarios in which treatment is beneficial to both the individual and to society or where antibiotics are detrimental to both the individual and society. In the former case antibiotic use should be encouraged, and in the latter it should be discouraged, but neither scenario has a conflict between the interests of the individual and society. However, if a resistant mutation is transmitted efficiently and occurs rarely during the course of treatment, the individual may receive much of the benefit of treatment, and the community may receive much of the harm. A tragedy of the commons can indeed result, although this is only one of several possible outcomes from a game theoretic standpoint (as shown in Figure 2). In general, outcomes depend on the frequency of a resistant organism occurring with treatment and the relative fitness of the resistant organism.

We note that the two models presented here have several limitations. Analyses are based on equilibrium comparative statics and assume perfect information. In reality, decisions would be made with partial information in real time, leading to a dynamic game theoretic problem [63]. We have also assumed a large population (so that stochastic effects may be neglected). Moreover, the model contains the following simplifying assumptions: lack of immunity, the absence of coinfection, a linear dependence of the force of infection on the prevalence fraction, the availability of only a single drug, and the assumption of a homogeneously mixing population in which network structure is ignored. Further studies which included these features could search for a conflict of interest on a case by case basis.

It has been estimated that one-third of antibiotic use in the United States is unnecessary [64]. Is this overuse harmful for society in all cases? The assumption has been that restrictions are beneficial because they reduce the prevalence of drug-resistance. Although our model clearly supports the view that antibiotic restriction may often be necessary, such restrictions are not beneficial in all cases. We have seen that community harm may result from individuals using antibiotics in a way that is not helpful to the individual themselves, but occur in some scenarios even when the individuals receive health benefits from using antibiotics. When individual incentives are in conflict with the well being of the community, fundamentally different ethical issues and policy tools are needed than when such conflicts do not exist. Understanding how such dilemmas may arise in specific antibiotic settings will require an improved empirical basis.

## Supporting Information

Text S1 **Details of selected calculations are provided in the Appendix (Text S1).**
(PDF)
