# Supplementary material for: When Does Overuse of Antibiotics Become a Tragedy of the Commons?
Source: PLoS One. 2012 Dec 7;7(12):e46505. doi: 10.1371/journal.pone.0046505 (PMC3517551; doi:10.1371/journal.pone.0046505)
Supplement: Text S1 — Details of selected calculations are provided in the Appendix (Text S1). (PDF) [file pone.0046505.s001.pdf]

Supporting Information for "When does overuse of antibiotics become a tragedy of the commons?", *PLoS One*

Travis C. Porco[1,2,3,7], Daozhou Gao[1], James C. Scott[4], Eunha Shim[5], Wayne T. Enanoria[1,3], Alison P. Galvani[6], and Thomas M. Lietman[1,2,7,8*]

1 Francis I. Proctor Foundation for Research in Ophthalmology, Box 0412, University of California, San Francisco, California USA
2 Department of Epidemiology and Biostatistics, University of California, San Francisco, San Francisco, California USA
3 Center for Infectious Disease and Emergency Readiness, University of California, Berkeley, Berkeley, California USA
4 Department of Mathematics and Statistics, Colby College, Waterville, Maine USA
5 Department of Epidemiology, Graduate School of Public Health, University of Pittsburgh, Pittsburgh, Pennsylvania USA
6 School of Medicine, Yale University, New Haven, Connecticut USA
7 Department of Ophthalmology, University of California, San Francisco, San Francisco, California USA
8 Institute for Global Health, University of California, San Francisco, San Francisco, California USA

* To whom correspondence should be directed: Thomas M. Lietman, F.I. Proctor Foundation, Department of Ophthalmology and Department of Epidemiology and Biostatistics, University of California, San Francisco, UCSF Box 0412, San Francisco, California 94143-0412 USA; email `tom.lietman@ucsf.edu`; phone (415) 502-2662; FAX (415) 476-0527.

September 19, 2012

Appendix

Model 1

For Table 1 in the main text, we computed the quantities A, B, C, and D using Equation (17). In this section, we first show that $B > A$ when $R_{SS} > 1$ and $R_{SS} > R_{RR}$, and then show that the result $B > A$ holds under the assumption $R_{RR} > R_{SS}$ as well, provided $R_{RR} < R_{0,SS}$. This includes the case where the drug-sensitive and drug-resistant organisms behave the same (except for transmission). Specifically, denoting the payoff for an individual as $P(\theta_i, \theta) = X^{(i)*}$, where θ_i is the treatment rate for the individual and θ the treatment rate for the community, we have $A = P(0,0) = \rho_S/\beta_S$, $B = P(0, \theta_{\max})$, $C = P(\theta_{i,\max}, 0)$, and $D = P(\theta_{i,\max}, \theta_{\max})$. In particular

$$B = \frac{\rho_S \rho_R}{\rho_S(\rho_R + \lambda_R^0) + \lambda_S^0 \rho_R}$$

where $\lambda_S^0(\theta)$ and $\lambda_R^0(\theta)$ are defined as in Equations (19) and (20), respectively. Then we have

$$B > A \Leftrightarrow \rho_R \beta_S > \rho_S(\rho_R + \lambda_R^0) + \lambda_S^0 \rho_R \Leftrightarrow \rho_R(\beta_S - \rho_S) > \rho_S \lambda_R^0 + \rho_R \lambda_S^0$$

$$\Leftrightarrow \rho_R(\beta_S - \rho_S)(\beta_S \rho_R + \beta_S \delta\theta - \beta_R \rho_S - \beta_R \theta) > (\rho_S \beta_R \delta\theta + \rho_R(\beta_S \rho_R - \beta_R \rho_S - \beta_R \theta))(\beta_S - \rho_S - \theta)$$

$$\Leftrightarrow \rho_R(\beta_S - \rho_S)\beta_S \delta\theta > \rho_S \beta_R \delta\theta(\beta_S - \rho_S - \theta) - \rho_R \theta(\beta_S \rho_R - \beta_R \rho_S - \beta_R \theta)$$

$$\Leftrightarrow \delta(\rho_R(\beta_S - \rho_S)\beta_S - \rho_S \beta_R(\beta_S - \rho_S - \theta)) + \rho_R(\beta_S \rho_R - \beta_R \rho_S - \beta_R \theta) > 0.$$

In fact, it follows $R_{SS} = \beta_S/(\rho_S + \theta) > 1$ and $R_{SS} = \beta_S/(\rho_S + \theta) > R_{RR} = \beta_R/\rho_R$ that

$$\rho_R \beta_S > \rho_S \beta_R \Rightarrow \rho_R \beta_S(\beta_S - \rho_S) > \rho_S \beta_R(\beta_S - \rho_S) > \rho_S \beta_R(\beta_S - \rho_S - \theta) > 0$$

and $\beta_S \rho_R > \beta_R \rho_S + \beta_R \theta$.

Moreover, an individual who chooses not to treat will benefit more from higher community treatment (i.e., $\partial B/\partial\theta > 0$) provided that $\rho_R - \delta\rho_S > 0$, $R_{SS} > 1$ and $R_{SS} > R_{RR}$. After some straightforward but lengthy algebraic calculations, we find that $\partial B/\partial\theta > 0$ is equivalent to

$$(\beta_S \rho_R - \beta_R(\rho_S + \theta))^2 + \frac{(\beta_S \rho_R - \beta_R \rho_S)^2}{\rho_R - \delta\rho_S} \beta_S \delta > \beta_R \beta_S \delta \theta^2.$$

It suffices to show that

$$\frac{(\beta_S\rho_R - \beta_R\rho_S)^2}{\rho_R - \delta\rho_S}\beta_S\delta > \beta_R\beta_S\delta\theta^2, \text{ or equivalently, } (\beta_S\rho_R - \beta_R\rho_S)^2 > (\rho_R - \delta\rho_S)\beta_R\theta^2.$$

If $0 < \rho_R - \delta\rho_S \le \beta_R$, then

$$R_{SS} > R_{RR} \Leftrightarrow \beta_S\rho_R - \beta_R\rho_S > \beta_R\theta \Leftrightarrow (\beta_S\rho_R - \beta_R\rho_S)^2 > \beta_R^2\theta^2 > (\rho_R - \delta\rho_S)\beta_R\theta^2.$$

However, if $\rho_R - \delta\rho_S > \beta_R$, then

$$R_{SS} > 1 \Rightarrow \beta_S\rho_R - \beta_R\rho_S > (\rho_S + \theta)\rho_R - \beta_R\rho_S > \rho_R\theta + \rho_S(\beta_R + \delta\rho_S) - \beta_R\rho_S > \rho_R\theta$$

and hence $(\beta_S\rho_R - \beta_R\rho_S)^2 > \rho_R^2\theta^2 > (\rho_R - \delta\rho_S)\beta_R\theta^2$.

We now assume that $R_{RR} > R_{SS}$ and $R_{RR} < R_{0,SS}$, where $R_{RR} = \beta_R/\rho_R$, $R_{SS} = \beta_S/(\rho_S + \theta)$ and $R_{0,SS} = \beta_S/\rho_S$ When $\theta_i = 0$ and $\theta = 0$, we have $\lambda_S^0(0) = \beta_S - \rho_S$ and $\lambda_R^0(0) = 0$. Hence $A = P(0,0) = \rho_S/\beta_S = 1/R_{0,SS}$. Also, if $\theta_i = 0$ and $\theta = \theta_{max} > \theta_{crit}$ (here θ_{crit} is the unique root of $R_{RR} = R_{SS}(\theta)$), we have $R_{RR} > R_{SS}(\theta_{max})$ and hence $\lambda_S^0(\theta_{max}) = 0$ and $\lambda_R^0(\theta_{max}) = \beta_R - \rho_R$. Thus, $B = P(0,\theta_{max}) = \dfrac{\rho_S\rho_R}{\rho_S(\rho_R + \lambda_R^0(\theta_{max}))} = \dfrac{\rho_R}{\beta_R} = 1/R_{RR}$. Therefore, $A < B$ is true if $R_{RR} > R_{SS}$ and $R_{RR} < R_{0,SS}$.

When $\rho_R - \delta\rho_S < 0$, we have a sufficiently long duration for resistant infection that it is always best for an individual not to be treated. We assume coexistence conditions $R_{SS} > 1$ and $R_{SS} > R_{RR}$, i.e. the community treatment rate θ being considered is not at a high enough rate to eradicate sensitive infection. In this case, individual incentives favor undertreatment.

Model 2

For the community dynamics of Model 2, the equilibrium fraction of individuals in each state for the coexistence equilibrium can be calculated explicitly. The fraction in the susceptible class is given by

$$\frac{X^*}{N} = \frac{(\tilde{\rho}_S + \tilde{\theta})(\gamma_S + \rho_S + \theta)}{\beta_S(\tilde{\rho}_S + \tilde{\theta}) + \tilde{\beta}_S\gamma_S} = \frac{1}{R_{SS}}.$$

When $R_{SS} < 1$, this equation yields a fraction greater than 1, which is impossible; the coexistence equilibrium cannot exist when $R_{SS} < 1$. Moreover, because this increases as θ increases, the higher the rate of treatment of the mild stage, the lower the prevalence of infection. (However, it is possible to increase the fraction in the severe state, as will be shown). For the fraction with mild infection with the drug-sensitive organism,

$$\frac{Y_S^*}{N} = \frac{c_1(\tilde{\rho}_S + \tilde{\theta})\big(\tilde{\beta}_S\gamma_S + (\tilde{\rho}_S + \tilde{\theta})(\beta_S - (\gamma_S + \rho_S + \theta))\big)}{c_2(\beta_S\tilde{\rho}_S + \beta_S\tilde{\theta} + \tilde{\beta}_S\gamma_S)} \tag{1}$$

where

$$c_1 = \tilde{\rho}_R(\tilde{\rho}_S + \tilde{\theta})\big(\beta_S(\gamma_R + \rho_R) - \beta_R(\gamma_S + \rho_S + \theta)\big) + \tilde{\beta}_S\tilde{\rho}_R\gamma_S(\gamma_R + \rho_R) - \tilde{\beta}_R\gamma_R(\tilde{\rho}_S + \tilde{\theta})(\gamma_S + \rho_S + \theta),$$

$$\begin{aligned} c_2 = {} & (\beta_S(\tilde{\rho}_S + \tilde{\theta}) + \tilde{\beta}_S\gamma_S)\big(c_2'(\gamma_R + \rho_R) + \delta\theta(\tilde{\rho}_S + \tilde{\theta})(\gamma_R + \tilde{\rho}_R)\big) \\ & - (\tilde{\rho}_S + \tilde{\theta})(\gamma_S + \rho_S + \theta)\big(\beta_R c_2' + \tilde{\beta}_R(\gamma_R(\gamma_S + \tilde{\rho}_S + \tilde{\theta}) - \tilde{\delta}\gamma_S\tilde{\theta})\big), \end{aligned}$$

and

$$c_2' = \gamma_S(\tilde{\delta}\tilde{\theta} + \tilde{\rho}_R) + \tilde{\rho}_R(\tilde{\rho}_S + \tilde{\theta}).$$

The fraction with mild infection with the drug-resistant organism may be expressed in terms of Y_S^*/N (Equation (1)):

$$\frac{Y_R^*}{N} = \frac{Y_S^*}{N}\frac{\beta_S\theta\delta\tilde{\rho}_R(\tilde{\rho}_S + \tilde{\theta}) + \tilde{\beta}_S\tilde{\rho}_R\delta\gamma_S\theta + \tilde{\beta}_R\tilde{\theta}\tilde{\delta}\gamma_S(\gamma_S + \rho_S + \theta)}{\tilde{\rho}_R(\gamma_R + \rho_R)\big(\beta_S(\tilde{\rho}_S + \tilde{\theta}) + \tilde{\beta}_S\gamma_S\big) - (\tilde{\rho}_S + \tilde{\theta})(\gamma_S + \rho_S + \theta)\big(\beta_R\tilde{\rho}_R + \tilde{\beta}_R\gamma_R\big)} \tag{2}$$

The fraction with severe infection due to the drug-sensitive organism is

$$\frac{\tilde{Y}_S^*}{N} = \frac{Y_S^*}{N}\frac{\gamma_S}{\tilde{\rho}_S + \tilde{\theta}}, \tag{3}$$

as given directly from Equation (**??**). Note that this ratio is independent of the treatment rate of mild infection.

Finally, we express the fraction with severe infection due to the drug-resistant organism in terms of $\frac{Y_S^*}{N}$:

$$\frac{\tilde{Y}_R^*}{N} = \frac{Y_S^*}{N}\frac{c_3}{c_1(\tilde{\rho}_S + \tilde{\theta})}. \tag{4}$$

where

$$c_3 = (\beta_S(\tilde{\rho}_S + \tilde{\theta}) + \tilde{\beta}_S\gamma_S)\big(\delta\theta\gamma_R(\tilde{\rho}_S + \tilde{\theta}) + \tilde{\theta}\tilde{\delta}\gamma_S(\gamma_R + \rho_R)\big) - \beta_R\tilde{\delta}\gamma_S\tilde{\theta}(\tilde{\rho}_S + \tilde{\theta})(\gamma_S + \rho_S + \theta).$$

The equilibrium force of infection for the drug-sensitive organism is then given by substituting Equations (1) and (3) into the following:

$$\lambda_S(\theta) = \beta_S\frac{Y_S^*}{N} + \tilde{\beta}_S\frac{\tilde{Y}_S^*}{N}. \tag{5}$$

Similarly, the equilibrium force of infection for the drug-resistant organism is given by substituting Equations (2) and (4) into the following:

$$\lambda_R(\theta) = \beta_R\frac{Y_R^*}{N} + \tilde{\beta}_S\frac{\tilde{Y}_R^*}{N}. \tag{6}$$

The overall fraction of time spent in the severe state for the community level analysis for Model 2 is then

$$V(\theta) = \frac{\tilde{Y}_S^* + \tilde{Y}_R^*}{N}. \tag{7}$$

The value of θ which minimizes $V(\theta)$ is the utilitarian optimum mild treatment level, i.e. the treatment rate which minimizes the fraction of severe disease for the population.
